# The assessment of the balance system in cranial artery stenosis

**DOI:** 10.1002/brb3.1695

**Published:** 2020-07-20

**Authors:** Karolina Dorobisz, Tadeusz Dorobisz, Tomasz Zatoński

**Affiliations:** ^1^ Department of Otolaryngology, Head and Neck Surgery Wroclaw Medical University Wroclaw Poland; ^2^ Department of Vascular Surgery Wroclaw Medical University Wroclaw Poland

**Keywords:** balance system, cranial artery stenosis, stroke, vertigo

## Abstract

**Introduction:**

Vertigo and balance disorders are a significant clinical problem, especially in elderly patients. The narrowing of cranial vessels may be asymptomatic or produce neurological symptoms. Very often nonspecific signs of ischemia occur, such as headache, vertigo, or dizziness.

**Objective:**

The objective of the study was to assess the effect of carotid and vertebral arteries stenosis on the function of the equilibrium organ on the basis of electronystagmography and posturography.

**Material:**

The study was conducted in 63 patients, presenting with carotid and vertebral arteries stenosis. The control group consisted of 32 healthy persons.

**Methods:**

All patients were subjected to precise audiological and otoneurological diagnostic examinations. Prior to being qualified for the study, patients were subjected to the assessment of arteries by means of Doppler ultrasonography. The vestibular organ was assessed by means of physical examination as well as by electronystagmography and posturography testing.

**Results and conclusions:**

The study revealed statistically significant reduction in the results of the equilibrium organ assessments in patients with carotid and vertebral arteries sclerosis as compared to the control group. Abnormal ENG records in the study group patients were observed particularly in the pendulum test, optokinetic test, and the assessment of positional nystagmus, possibly indicating disturbances within the central part of the equilibrium system. Disturbed blood flow in arteries had also an important impact on spinovestibular reflexes and resulted in disturbed postural stability control. On the basis of the conducted studies, it is concluded that diagnostic examinations for carotid and vertebral artery stenosis should be performed in patients with equilibrium system disorders.

## Graphical abstract

The study revealed statistically significant reduction in the results of the equilibrium organ assessments in patients with carotid and vertebral arteries sclerosis as compared to the control group. On the basis of the conducted studies, it is concluded that diagnostic examinations for carotid and vertebral artery stenosis should be performed in patients with equilibrium system disorders.

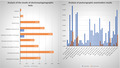

## 1 INTRODUCTION

Vertigo and balance disorders are a significant clinical problem, especially in elderly patients (Hannaford et al., 2005; Maarsingh et al., 2010; Yardley, Owen, Nazareth, & Luzon, 1998). They occur in around 30% of people over the age of 65 and in 50% of those over 80 (Hannaford et al., 2005). Diagnosis and treatment of patients with balance disorders require vast knowledge and experience as well as multidisciplinary and multidirectional diagnostic actions.

Emboli of the cerebral arteries, including internal carotid arteries and vertebral arteries, are the most frequent among the causes of dizziness of the central origin with the ischemic nature (Dietrich, 2002).

The narrowing of cranial vessels may be asymptomatic or produce typical neurological symptoms. Very often, however, nonspecific signs of ischemia occur, such as headache, vertigo, or dizziness (Seemungal, 2007; Terzi, Arslanoglu, Demiray, Eren, & Cancuri, 2015). They may indicate clinically relevant stenosis of the cranial arteries, mainly in the area of the vertebral artery and internal carotid artery, as well as the risk of ischemic stroke. Dizziness may be the only symptom of TIA in the vertebral‐basilar system, in the course of subclavian steal syndrome, chronic dysfunction of the vertebral‐basilar system, ischemic brain stem and cerebellum syndrome, and cerebellar hemorrhage (Gutmann, Wollenberg, Krampert, & Mees, 1993; Mees, Gutmann, & Wollenberg, 1992).

The available scientific reports do not accurately explain the correlation between dizziness and stenosis of the cranial arteries.

The aim of the study was to assess the impact of cranial artery stenosis on the function of the equilibrium system based on ENG and posturographic examination. The goal was achieved by answering the following questions:

Do the parameters of assessment of spontaneous and induced nystagmus recorded in ENG differ in the group of patients with narrowing of the cranial arteries compared with the group of persons without stenosis?

Do the parameters of vestibulospinal reflexes, recorded in posturographic examination, differ in the group of patients with narrowing of the head arteries from those in the group of persons without stenosis?

## 2 MATERIAL

### 2.1 Control group

The control group consisted of 32 healthy persons (14 men, 18 women) aged from 48 to 75 years (mean age *M* = 61, standard deviation *SD* ± 7 years). People qualified to this group did not report any middle ear disorders, hearing problems, dizziness, or balance disturbances; no pathology was found on the otolaryngological examination; the tympanic membrane was normal in the otoscopic test.

In persons from this group, an ultrasound examination of the cranial arteries was performed, the result of which was normal. The medical history excluded cardiovascular diseases.

### 2.2 Study group

The study included 63 patients (32 men, 31 women) aged from 45 to 75 years (mean age *M* = 62.6, standard deviation *SD* ± 7.4 years). These were patients with stenosis of the cranial arteries, referred to the Department of Vascular Surgery of the University Clinical Hospital in Wrocław in the years 2014–2015. The patients were divided into two age groups (45–60 years, 61–75 years). The study excluded patients with previous cranial trauma, meningitis, and neurological disorders (including epilepsy, multiple sclerosis, and Parkinson's disease). The analysis did not take into account patients receiving ototoxic medications, with the history of hearing and balance problems, as well as subjects with ENT abnormalities. During the study, none of the patients received medications that could affect the nervous system. All subjects were a group homogeneous in terms of the degree of cranial artery stenosis. Patients with internal carotid artery stenosis between 70% and 90% or with a peak systolic velocity of the vertebral artery flow above 120 cm/s were qualified to the study group.

The examined patients were classified into the following groups: patients with unilateral vertebral artery stenosis (*n* = 23), patients with unilateral carotid artery stenosis (*n* = 28), and patients with bilateral internal carotid artery stenosis (*n* = 12).

In the study group, 48 (76.2%) patients reported symptoms of cranial artery stenosis. 18 (28.6%) patients reported neurological symptoms, while in 45 (71.4%) patients, narrowing of the head arteries coexisted with ENT symptoms. Among the ENT symptoms, 45 (71.4%) patients noticed vertigo. In the group of patients with vertebral artery stenosis, four (17.3%) patients reported neurological symptoms, and ENT 20 (86.9%). In this subgroup, vertigo occurred in up to 20 (86.9%) patients. In the group of patients with internal carotid artery stenosis, 14 (40%) patients had neurological symptoms, and ENT in 25 (62.5%). 18 (45%) patients suffered from vertigo.

## 3 METHOD

All patients were subjected to thorough otoneurological diagnostic procedures. In the first stage, a detailed medical history was taken. It covered the main ailment, chronic and past diseases with particular emphasis on the pathology of the middle and inner ear and the presence of any complications of the main disease, as well as conditions that may affect the hearing and balance organs, dizziness, and balance disorders. Attention was paid to the presence of symptoms that could indicate vestibular‐cochlear organ disorders, such as headache, unstable body posture, problems with daily activities, or the loss of independence in performing basic activities. The assessment also included the risk factors of damage to the hearing and balance organ by exposure to the noise at work or as a result of taking ototoxic drugs.

Each patient was consulted by a neurologist and ophthalmologist.

Prior to qualifying to the study, the cranial arteries were evaluated using Doppler ultrasonography.

The otolaryngological examination was performed in each patient.

The vestibular organ was assessed in the physical examination, including Romberg's, Unterberger's, and Babiński–Weill's tests. In the next stage, objective tests, such as posturographic and electronystagmographic (ENG) examinations, were carried out.

The ENG examination was carried out with the help of a Hartmann two‐channel computer electronystagmograph. The study assessed the occurrence of nystagmus, which is an objective symptom of vestibular disorders. The registered eye movements were perceived by electrodes located on the forehead and temples.

Electronystigmographic examination recorded:

- spontaneous nystagmus with eyes open in a sitting position,
- spontaneous nystagmus with eyes closed in a sitting position,
- the presence of positional nystagmus in four positions of the body—on the back, on the left side, on the right side, in the Rose position (on the back with the head tilted back), as well as when performing Hallpike maneuvers to the right and left. Positional nystagmus was assessed by Nylene classification: type I—nystagmus changing direction in different head positions, type II—nystagmus with a constant direction regardless of the position of the head, type III—nystagmus changing irregularly direction and amplitude in the same head position,
- optokinetic nystagmus to the right and left. The record was rated as symmetrical (correct) or asymmetrical based on the course and average velocity of the free phase of nystagmus,
- pendulum tracking test performed by observing a light point moving at a frequency of 0.4 Hz and an amplitude of 30 degrees. The recording lasted 30 s. Symmetries and correctness of recording were assessed as normal (I type), distorted (II type), and completely distorted (III type),
- caloric tests were performed by the Fitzgerald–Hallpike method. The test was carried out using water at 30 and 44°C. The irrigation time was 30 s. Nystagmus assessment was performed by analyzing by the computer system the frequency of nystagmus deflections in selected 30 s of the peak of reaction. The Jongkees formula was used to assess nystagmus symmetry.

The following norms for caloric test parameters have been adopted:

- unilateral weakness – <25%,
- directional advantage – <30%,
- baseline shift (nystagmus present before caloric testing) – <6 degrees/s,
- bilateral weakness – >12 degrees/s,
- overactivity – <140 degrees/s,
- attenuation with fixation – <60%.

The ENG record also evaluated the presence of square waves, dysrhythmia, and dysmetry.

Vestibulospinal reflex tests were performed using a VSR Basic Balance Master posturograph from NeuroCom International Inc. The strength plate was calibrated to the height of the subject, and the patient's center of gravity was presented on the monitor screen. The study was preceded by registration of the study age, weight, and height. During the tests, the patient stood upright, his arms along the torso. Body posture control was assessed by performing five tests:

1. Weight Bearing Squat – WBS

During the test, the patient stood upright with his eyes directed straight ahead. Measurements of the percentage load on both legs were made in an upright position (0 degrees) and with the knees bent by 30, 60, and 90 degrees.

2. The modified Clinical Test for the Sensory Interaction on Balance (mCTSIB)

In a standing, stationary position, four tests were performed: with eyes open (EO) and closed (EC) standing on hard ground (FIRM) and with eyes open and closed standing on a soft surface ‐ sponge (FOAM). Each test was performed three times and each test lasted 10 s. The mean velocity of the center of gravity deflection was evaluated.

3. Unilateral Stance Test—US

The patient stood on the right, then on the left leg with eyes open and closed. The patient performed each of the trials three times. The mean velocity of the center of gravity deflections in degrees per second was analyzed.

4. Limits of Stability (LOS) test

During the test, the patient saw his marked center of gravity on the monitor screen, which he had to keep in the center. After the sound signal, the patient had to move his center of gravity to the target point in the shortest time and by the straightest way. Eight directions were studied—front, back, left, right, and four diagonals. Recorded were as follows:

response time (RT)—time from the moment of visual and acoustic signal to the beginning of the movement,
center of gravity swivel speed (MVL) to first end point (EPE),
direction control (DCL), that is, the comparison of the number of moves toward the target compared with the number of incorrect moves in percentage,
final intended swing in percentage (MXE).

5. Rhythmic center of gravity transfer test (RWS)

The patient shifted his center of gravity sideways (L‐P) or forward–backward (P‐T), the tilting rhythm imposed the cursor on the screen moving at a speed of 1/s, 2/s, 3/s. The mean velocity of the center of gravity movement in degrees per second and the direction control value (DCL) resulting from the comparison of the number of movements were evaluated toward the goal to uncoordinated movements in percentage.

All procedures performed in studies involving human participants were in accordance with the ethical standards of the institutional and national research committee and with the 1964 Helsinki declaration and its later amendments or comparable ethical standards. The study was accepted by ethics committee in Wroclaw Medical University.

## 4 RESULTS

### 4.1 The analysis of electronystagmographic examination results

The ENG showed many abnormalities in patients with cranial artery stenosis. Spontaneous nystagmus with closed eyes was reported in 20 (31.8%) patients from the study group, while none of the persons from the control group demonstrated nystagmus, this difference was significant (*p* < 0.05). In the study group, positional nystagmus was observed in 33 (52.4%) patients, with the majority of Nylen I (28.6%) and Nylen III type nystagmus (22.2%). Abnormal optokinetic nystagmus was significantly more frequently (*p* < 0.05) observed in the study group compared with the control group (32 patients—50.8% vs. two patients—6.3%). An abnormal record was also significantly more often (*p* < 0.05) observed in the pendulum test in patients from the study group compared with the control group (24 vs. two persons). Square waves were statistically significantly more frequently (*p* < 0.05) reported in the ENG record in patients with cranial artery stenosis compared the control group (19 vs. 0). These data are illustrated in Table 1.

**TABLE 1:** The results of labyrinth tests (ENG) obtained in the study group compared with the control group

	Total *N* = 95		Test group *N* = 63		Control group *N* = 32		*B* versus *K* *p*
Spontaneous nystagmus:	*N*	%	*N*	%	*N*	%	
No	75	78.9%	43	68.2%	32	100.0%	**<0.001**
Yes	20	21.1%	20	31.8%	0	0.0%	
44°C SPV [°/s] – RE—right ear							
*M* ± *SD*	5.27 ± 3.68		5.00 ± 4.36		5.81 ± 1.61		**0.012**
*Me* (*Q*_1_; *Q*_3_)	5.5 (3.4; 6.6)		4.3 (2.1; 6.5)		6.2 (4.8; 7.2)		
44°C SPV [°/s] – LE—left ear							
*M* ± *SD*	−5.51 ± 3.89		−5.49 ± 4.42		−5.56 ± 2.64		0.230
*Me* (*Q*_1_; *Q*_3_)	−4.8 (−7.4; −3.2)		−4.3 (−8.1; −2.7)		−4.9 (−6.3; −3.9)		
|UP| versus |UL| (test Wilcoxon)	*p* = 0.621		*p* = 0.173		*p* = 0.217		×
30°C SPV [°/s] – RE							
*M* ± *SD*	−6.20 ± 3.17		−5.99 ± 3.38		−6.62 ± 2.72		0.180
*Me* (*Q*_1_; *Q*_3_)	−5.8 (−8.1; −3.5)		−4.8 (−8.2; −3.1)		−5.9 (−7.5; −4.8)		
30°C SPV [°/s] – LE							
*M* ± *SD*	5.73 ± 3.76		5.89 ± 4.12		5.41 ± 2.95		0.810
*Me* (*Q*_1_; *Q*_3_)	4.7 (2.9; 8.4)		4.9 (2.2; 8.9)		4.3 (3.5; 7.5)		
|RE| versus |LE|	*p* = 0.054		*p* = 0.866		***p* = 0.003**		×
Positional nystagmus:	*N*	%	*N*	%	*N*	%	**<0.001**
Absence	62	65.3%	30	47.6%	32	100.0%	**<0.001**
Nylen 1	18	18.9%	18	28.6%	0	0.0%	
Nylen 2	1	1.1%	1	1.6%	0	0.0%	
Nylen 3	14	14.7%	14	22.2%	0	0.0%	
Optokinetic nystagmus:	*N*	%	*N*	%	*N*	%	
Normal	61	64.2%	31	49.2%	30	93.8%	**<0.001**
Abnormal	34	35.8%	32	50.8%	2	6.3%	
Pendulum test:	*N*	%	*N*	%	*N*	%	
Normal	69	72.6%	39	61.9%	30	93.8%	**0.004**
Abnormal. type I	24	25.3%	22	34.9%	2	6.3%	
Abnormal. type II	2	2.1%	2	3.2%	0	0.0%	
Dysmetry:	*N*	%	*N*	%	*N*	%	
No	94	98.9%	62	98.4%	32	100.0%	1.000
Yes	1	1.1%	1	1.6%	0	0.0%	
Square waves:	*N*	%	*N*	%	*N*	%	
No	76	80.0%	44	69.8%	32	100.0%	**<0.001**
Yes	19	20.0%	19	30.2%	0	0.0%	
Dysrhythmia:	*N*	%	*N*	%	*N*	%	
No	92	96.8%	60	95.2%	32	100.0%	0.548
Yes	3	3.2%	3	4.8%	0	0.0%	

Significance means that *p* is less than or equal to .05.

The analysis of the ENG results in patients from the study group did not show statistically significant differences depending on the age and the type of narrowed artery.

### 4.2 Analysis of posturography results

The analysis of posturographic parameters in the study group demonstrated many statistically significant differences in comparison with the control group. Abnormal parameters in most posturographic tests were reported in the group of patients with cranial artery stenosis. The abnormalities were observed in the modified Clinical Test for the Sensory Interaction on Balance (mCTSIB) in which the incorrect speed of the center of gravity tilt was statistically significantly (*p* < 0.05) more frequently recorded in the study group in patients with closed eyes on the hard surface and with open and closed eyes on the soft surface compared with healthy people. The abnormal mean speed of the center of gravity tilt and the abnormal difference in mean speed of the center of gravity between the limbs in the Unilateral Stance Test (US) in patients with stenosis of the cranial arteries were recorded with open and closed eyes. In the Limits of Stability Test (LOS), compared with the control group, patients from the study group statistically significantly more often showed (*p* < 0.05) abnormalities in movement velocity (MVL) results when tilting backward and in the directional control (DCL) values when moving sideways and backward, just like in the case of the endpoint excursion (EPE) and maximum excursion (MXE) values. Compared with the control group, the evaluation of the Rhythmic Weight Shift Test (RWS) demonstrated statistically significant (*p* < 0.05) abnormalities in the results of the mean speed of the center of gravity tilt sideways at the rhythm of 3/s and when moving forward and backward at the rhythm of 2/s and 3/s. Compared with healthy individuals, in the RWS test, the results of the analysis of mean DCL values were more often statistically significantly (*p* < 0.05) abnormal in patients with cranial artery stenosis. These data are illustrated in Table 2.

**TABLE 2:** The results of posturographic tests obtained in the study group compared with the control group (number and proportion of abnormal results)

Test	Test group *N* = 63		Control group *N* = 32		*p*
	*n*	%	*n*	%	
1. Test Weight Bearing Squat (WBS)					
Normal results	55	87.3%	32	100.0%	0.109
Abnormal results‐left leg	5	7.9%	0	0.0%	
Abnormal results‐right leg	3	4.8%	0	0.0%	
Abnormal results:					
Test MCTSIB FIRM EO‐eyes open	3	4.8%	0	0.0%	0.548
Test MCTSIB FIRM EC‐eyes closed	38	60.3%	1	3.1%	**<0**.**001**
Test MCTSIB FOAM EO	19	30.2%	3	9.4%	**0**.**038**
Test MCTSIB FOAM EC	49	77.8%	9	28.1%	**<0**.**001**
Test UL L‐EO	5	7.9%	0	0.0%	0.164
Test UL R‐EO	8	12.7%	0	0.0%	**0**.**048**
Test UL L‐EC	39	61.9%	5	15.6%	**<0**.**001**
Test UL R‐EC	41	65.1%	6	18.8%	**<0**.**001**
Test LOS RT‐reaction time, forward	3	4.8%	0	0.0%	0.548
Test LOS RT backward	7	11.1%	0	0.0%	0.091
Test LOS RT right	7	11.1%	0	0.0%	0.091
Test LOS RT left	4	6.3%	0	0.0%	0.297
Test LOS MVL (°/s) forward	2	3.2%	0	0.0%	0.548
Test LOS MVL (°/s) backward	15	23.8%	0	0.0%	**0**.**002**
Test LOS MVL (°/s) right	4	6.3%	0	0.0%	0.297
Test LOS MVL (°/s) left	7	11.1%	0	0.0%	0.091
Test LOS DCL (%) forward	7	11.1%	0	0.0%	0.091
Test LOS DCL (%) backward	33	52.4%	2	6.2%	**<0**.**001**
Test LOS DCL (%) right	19	30.2%	0	0.0%	**<0**.**001**
Test LOS DCL (%) left	19	30.2%	0	0.0%	**<0**.**001**
Test LOS EPE (%) forward	13	20.6%	0	0.0%	**0**.**004**
Test LOS EPE (%) backward	27	42.9%	0	0.0%	**<0**.**001**
Test LOS EPE (%) right	16	25.4%	0	0.0%	**0**.**001**
Test LOS EPE (%) left	17	27.0%	0	0.0%	**<0**.**001**
Test LOS MXE (%) forward	16	25.4%	0	0.0%	**0**.**001**
Test LOS MXE (%) backward	34	54.0%	3	9.4%	**<0**.**001**
Test LOS MXE (%) right	20	31.7%	1	3.1%	**0**.**001**
Test LOS MXE (%) left	22	34.9%	1	3.1%	**<0**.**001**
Left–right					
Test RWS 1/S (°/s)	3	4.8%	0	0.0%	0.548
Test RWS 2/S (°/s)	6	9.5%	0	0.0%	0.093
Test RWS 3/S (°/s)	16	25.4%	0	0.0%	**0**.**001**
Test RWS DCL 1/S (%)	4	6.3%	0	0.0%	0.297
Test RWS DCL 2/S (%)	18	28.6%	2	6.2%	**0**.**015**
Test RWS DCL 3/S (%)	30	47.6%	5	15.6%	**0**.**003**
Forward–backward					
Test RWS 1/S (°/s)	4	6.3%	0	0.0%	0.297
Test RWS 2/S (°/s)	10	15.9%	0	0.0%	**0**.**015**
Test RWS 3/S (°/s)	18	28.6%	1	3.1%	**0**.**003**
Test RWS DCL 1/S (%)	14	22.2%	0	0.0%	**0**.**002**
Test RWS DCL 2/S (%)	36	57.1%	2	6.2%	**<0**.**001**
Test RWS DCL 3/S (%)	44	69.8%	7	21.9%	**<0**.**001**

Significance means that *p* is less than or equal to .05.

In the study group, the analysis of posturography results did not show statistically significant differences depending on the age of patients.

Patients with internal carotid stenosis achieved statistically significantly (*p* < 0.05) worse results in the US test and the LOS test compared to patients with vertebral artery stenosis.

Patients from the study group with the positive history of vertigo achieved statistically significantly (*p* < 0.05) worse results in all tests compared with the patients from the study group without vertigo (Table 3).

**TABLE 3:** The comparison of posturography results obtained in patients from the study group with and without dizziness (number and proportion of abnormal results)

Test	Patients without vertigo *N* = 18		Patients with vertigo *N* = 45		*p*
	*n*	%	*N*	%	
1. WBS					
Normal	17	94.4%	38	84.4%	0.466
Abnormal results‐left leg	1	5.6%	4	8.9%	
Abnormal result‐right leg	0	0.0%	3	6.7%	
Abnormal results:					
Test mCTSIB FIRM EO	28	62.2%	16	88.9%	1.000
Test mCTSIB FIRM EC	2	11.1%	36	80.0%	**<0**.**001**
Test mCTSIB FOAM EO	2	11.1%	17	37.8%	0.066
Test mCTSIB FOAM EC	9	50.0%	40	88.9%	**0**.**002**
Test US L‐EO	1	5.6%	4	8.9%	1.000
Test US R‐EO	2	11.1%	6	13.3%	1.000
Test US L‐EC	4	22.2%	35	77.8%	**<0**.**001**
Test US R‐EC	5	27.8%	36	80.0%	**<0**.**001**
Test LOS forward	0	0.0%	3	6.7%	0.551
Test LOS backward	1	5.6%	6	13.3%	0.347
Test LOS right	1	5.6%	6	13.3%	0.662
Test LOS left	0	0.0%	4	8.9%	0.317
Test LOS MVL (°/s) forward	0	0.0%	2	4.4%	1.000
Test LOS MVL (°/s) backward	2	11.1%	13	28.9%	0.195
Test LOS MVL (°/s) right	0	0.0%	4	8.9%	0.317
Test LOS MVL (°/s) left	1	5.6%	6	13.3%	0.662
Test LOS DCL (%) forward	1	5.6%	6	13.3%	0.662
Test LOS DCL (%) backward	6	33.3%	27	60.0%	0.102
Test LOS DCL (%) right	2	11.1%	17	37.8%	0.066
Test LOS DCL (%) left	2	11.1%	17	37.8%	0.069
Test LOS EPE (%) forward	1	5.6%	12	26.7%	0.087
Test LOS EPE (%) backward	3	16.7%	24	53.3%	**0**.**011**
Test LOS EPE (%) right	1	5.6%	15	33.3%	**0**.**026**
Test LOS EPE (%) left	2	11.1%	15	33.3%	0.115
Test LOS MXE (%) forward	1	5.6%	15	33.3%	**0**.**026**
Test LOS MXE (%) backward	5	27.8%	29	64.4%	**0**.**012**
Test LOS MXE (%) right	1	5.6%	19	42.2%	**0**.**006**
Test LOS MXE (%) left	2	11.1%	20	44.4%	**0**.**018**
Left–right					
Test RWS 1/S (°/s)	1	5.6%	2	4.4%	1.000
Test RWS 2/S (°/s)	1	5.6%	5	11.1%	0.664
Test RWS 3/S (°/s)	1	5.6%	15	33.3%	**0**.**026**
Test RWS DCL 1/S (%)	1	5.6%	3	6.7%	1.000
Test RWS DCL 2/S (%)	1	5.6%	17	37.8%	**0**.**013**
Test RWS DCL 3/S (%)	3	16.7%	27	60.0%	**0**.**002**
Forward–backward					
Test RWS 1/S (°/s)	1	5.6%	3	6.7%	1.000
Test RWS 2/S (°/s)	1	5.6%	9	20.0%	0.257
Test RWS 3/S (°/s)	1	5.6%	17	37.8%	**0**.**013**
Test RWS DCL 1/S (%)	1	5.6%	13	28.9%	0.051
Test RWS DCL 2/S (%)	5	27.8%	31	68.9%	**0**.**005**
Test RWS DCL 3/S (%)	7	38.9%	37	82.2%	**0**.**002**

Significance means that *p* is less than or equal to .05.

## 5 DISCUSSION

Inner ear disorders are very often overlooked in the diagnosis in the elderly, both by patients and doctors. Dizziness or vertigo is usually due to minor causes; patients experience a decrease in the quality of life, but, there is no risk of complications that may lead to further loss of health or death.

Benign positional dizziness is the most common cause of vertigo and balance disturbances (Kroenke, Hoffman, & Einstadter, 2000). Vertigo of the vascular origin is a very important clinical problem. Even though it is the second most common cause of all cases of vertigo (10%), it is a threat to the health and life of the patient. Although hearing loss, tinnitus, and vertigo may be the only symptoms preceding stroke (Seemungal, 2007), the problem of the impact of flow disturbances in the cranial arteries on the inner ear has not been thoroughly examined so far.

In our study, the assessment of the balance system disorders was based on the examinations which are a gold standard in dealing with these conditions. The balance system was evaluated on the basis of ENG and posturography. These tests require experience and the appropriate approach of technical staff and physicians to patients.

Diagnosis of the balance system based on a detailed medical history and careful ENT, neurological and ophthalmological examination, supplemented with the ENG and posturography is crucial and is the standard of management in patients with vertigo and balance disorders. Although the ENG is often burdened with a large number of artifacts, in combination with the posturography, it gives a full picture of the etiology of the disease, and thus allows for proper diagnosis and treatment.

The presence of vertigo in almost all patients with vertebral artery stenosis (86.9%) and almost every other patient with internal carotid stenosis is significant in the analyzed study results.

Our research shows that if we suspect cranial artery stenosis, both in the vertebral artery and internal carotid artery, asking questions about laryngological symptoms is an important element. A similar view is presented by Melliere, Le Chevillier, Ecollan, and Fitoussi (1992) and Terzi et al. (2015) who described otoneurological disorders in patients with cerebral ischemia caused by cranial artery stenosis. Kaźmierski, Kasielska, Bogusiak, Łysakowski, and Stelągowski (2012) found that up to 90% of patients with internal carotid artery stenosis felt vertigo, while tinnitus was experienced by about 80% of patients.

On physical examination, the patients from the control group did not show any abnormalities, while 60% of patients from the study group with vertebral artery stenosis demonstrated incorrect results in the Romberg's test, and 30% had spontaneous nystagmus. In the group of patients with internal carotid artery stenosis, 60% had an abnormal Romberg's test result; however, none of them had nystagmus. The results of basic and easy to examine symptoms confirm the relationship between cerebral blood flow disorders in the course of cranial artery stenosis and balance disturbances. Similar observations were made by Eckstein et al. (2008) and Melliere et al. (1992), showing that patients with the impaired blood flow in the cranial arteries also demonstrated postural disorders. Findings obtained by numerous authors and our research indicate that patients with this type of abnormalities should be examined for cerebral blood flow and inner ear disorders.

A comparative analysis of the ENG results showed significant differences between the study and the control group. Spontaneous nystagmus was recorded in the ENG in 31.8% of patients from the study group, but was not reported in any of the control group patients. Amor Dorado, Rubio Rodríguez, Costa, Juiz, and Rossi (2003), however, found that 15% of healthy patients over the age of 64 had spontaneous nystagmus.

Statistically significant (*p* < 0.05) differences between the study and control group were also found in the examination of positional nystagmus, optokinetic nystagmus, in the pendulum test and the test for square waves. Positional nystagmus Nylen 1 type was observed in 18 (28.6%) patients from the study group, Nylen 2 type was registered in one (1.6%) patient, while positional nystagmus Nylen 3 type was reported in 14 (22.2%) patients from the study group. The ENG did not reveal positional nystagmus in any patients from the control group. Despite studies, the mechanism in which positional nystagmus develops could not be fully explained (Brandt, 1990). Abnormal optokinetic nystagmus was reported in 32 (50.8%) patients from the study group and in two subjects (6.3%) from the control group. Many authors have described the distortion of optokinetic nystagmus, also of the vascular origin, in damage to the central nervous system. The abnormal result of the pendulum test was recorded in 24 (38%) patients from the study group and in two (6.3%) subjects from the control group. Square waves were also significantly statistically (*p* < 0.05) more frequently observed in patients from the study group; they were found in 19 (30.2%) patients with cranial artery stenosis. Square waves were not reported in the ENG in any patient from the control group. In addition, dysmetry (in one patient) and dysrhythmia (in three patients) were detected in patients with cranial artery stenosis. These test results indicate significantly more frequent disorders of the central origin in the ENG, which is associated with cerebral ischemia caused by atherosclerosis and clinically relevant stenosis of the cranial arteries. Similar observations were made by other researchers (Grad & Baloh, 1989). According to the authors quoted above, this proves multilevel damage to the balance system.

There were no statistically significant differences in ENG results between patients with vertebral artery stenosis compared to subjects with internal carotid artery stenosis, but spontaneous nystagmus was more common in patients with vertebral artery stenosis (39.1% vs. 27.5%). However, the presence of positional nystagmus was more frequently recorded in patients with internal carotid stenosis (type Nylen 1 32.5% vs. 21.7%; type Nylen 3 27.5% vs. 13%), but these were not statistically significant differences. In the group of patients with internal carotid artery stenosis, an abnormal record in the pendulum test and square waves were more frequent. These results suggest an advantage of balance disorders in the vestibular organ in patients with vertebral artery stenosis, while in subjects with internal carotid artery stenosis, abnormalities of the central‐neurological origin are more often. It is significant the patients with internal carotid artery stenosis manifest disorders in the vestibular organ; however, central damage is much more pronounced.

Spontaneous nystagmus was more common (37.8% vs. 16.7%) in the presence of otolaryngological symptoms. Interestingly, there was no statistically significant relationship between the presence of vertigo and ENG results, which may indicate a subclinical course of the disease or adaptation of patients to the condition.

The comparison of posturography results between the study and control group revealed abnormalities in the mCTSIB, US, LOS, and RWS test. The WBS test demonstrated almost similar body weight distribution on each of the lower limbs. According to Broussard, DeRocher, and Kalkofen (1996), the load on the lower limbs is almost similar and the differences do not exceed 7%.

The abnormal mean speed of the center of gravity tilt in the mCTSIB test was significantly (*p* < 0.05) more often recorded in the study group of patients on the hard and soft surface with the visual control turned off. The limitation of prioprioreceptive information while maintaining visual control also significantly (*p* < 0.05) worsened the stability of patients with stenosis of the cranial arteries compared to the results obtained in healthy people. With the use of all sensory information (open eyes, hard surface), postural stability in patients from the study group did not statistically significantly differ compared with the subjects from the control group.

Patients with cranial artery stenosis had a great difficulty in another test performed to assess the ability to maintain postural stability under static conditions. The abnormal mean speed of the center of gravity tilt and the abnormal difference in mean speed of the center of gravity between the limbs were reported in the US test in patients from the study group mainly when testing with closed eyes, that is, after turning off the visual control.

The LOS test, which is used to evaluate the balance system under dynamic conditions, showed statistically significant (*p* < 0.05) abnormalities in the study group in the MVL test with the body directed backward, in the DCL in direction to the right, left and the body tilted backward, in the EPE values in all tilt directions and in the MXE values in all directions. In their study, Geldhof et al. (2006) also indicate the weakest control of postural stability when tilting backward. This is most likely due to the lack of adaptation of bones and joints to backward tilts. According to literature data, the speedy reaction and precise body movements allow for maintaining balance under dynamic conditions (Norre, 1993; Riach & Starkes, 1993). The correct result of the LOS test depended on properly functioning visual coupling. The patient's reaction time to a command to move was an important element of the LOS test which depended mainly on higher nervous functions (Hirabayashi & Iwasaki, 1995). Although in patients from the study group, the reaction time was delayed compared with subjects from the control group, this difference was not statistically significant.

In the RWS test, in patients with cranial artery stenosis, significant (*p* < 0.05) abnormalities were found in the mean speed of the center of gravity tilt compared with healthy individuals, at the rhythm of tilts 3/s from side to side and when moving forward–backward at the rhythm of 2/s and 3/s. Mean DCL values were also significantly (*p* < 0.05) more often abnormal in patients from the study group when tilting left–right at the rhythm of 2/s and 3/s and forward–backward at the rhythm of 1/s, 2/s, and 3/s. Patients from the study group achieved the worst results with velocity of 3/s forward–backward. Abnormal motor coordination in patients with cranial artery stenosis most likely results from damage to information processing in the central nervous system.

The results indicate a significant damage to the balance system in the group of patients with cranial artery stenosis compared to the control group.

Patients with internal carotid artery stenosis achieved statistically significantly (*p* < 0.05) worse results compared to subjects with vertebral artery stenosis in the US test with closed eyes and in the LOS test, especially in DCL values. This may indicate more severe damage to postural control in a group of patients with internal carotid artery stenosis. There is no discussion on the subject in literature; however, with other conditions, such as multiple sclerosis and Parkinson's disease, significant differences in posturography results are also recorded.

## 6 LIMITATIONS OF THE STUDY

The study has not been randomized. The group of patients was relatively small. That was due to narrow study inclusion criteria. A full package of otoneurologic examinations comprised a study cycle lasting approximately 3 hr. A need for patients to focus on the study and to cooperate with a technician in charge of the examinations gave rise to the situation, in which some of the patients were not able to finish their study cycle. Therefore, they must have been excluded from the study group. The following were indicated: follow‐up examinations, a new series of examinations to check the repeatability of the results, and—on the occasion of the removal of obliteration in carotid arteries—a postoperative follow‐up visit.

## 7 CONCLUSIONS

1. A decrease in the cranial artery flow causes balance disturbances, especially in the central part, as evidenced by the ENG abnormalities in the pendulum test, the optokinetic test, and the evaluation of positional nystagmus.
2. Flow disturbances in the cranial arteries have a significant influence on vestibular‐spinal reflexes, causing problems in the postural stability control.
3. Abnormalities in electronystagmographic and posturographic examinations in patients with cranial artery stenosis are not always clinically manifested.
4. Diagnostic examinations for carotid and vertebral artery stenosis should be performed in patients with equilibrium system disorders.

## CONFLICT OF INTERESTS

Authors declare that they have no conflict of interest.

## AUTHOR CONTRIBUTION

Karolina Dorobisz conceived of the presented idea. Karolina Dorobisz and Tadeusz Dorobisz carried out the experiment. Karolina Dorobisz wrote the manuscript with support from Tadeusz Dorobisz and Tomasz Zatonski. Tomasz Zatonski supervised the project. All authors discussed the results and contributed to the final manuscript.

## Data Availability

All data analyzed during this study are included in this published article.
